# Rate and Risk Factors of Acute Myocardial Infarction after Debut of Chronic Kidney Disease—Results from the KidDiCo

**DOI:** 10.3390/jcdd9110387

**Published:** 2022-11-09

**Authors:** Jan Dominik Kampmann, James Goya Heaf, Christian Backer Mogensen, Sofie Ronja Petersen, Donna Lykke Wolff, Hans Mickley, Frans Brandt

**Affiliations:** 1 Department of Internal Medicine, Hospital of Southern Jutland, Sønderborg, Sydvang 1, 6400 Sønderborg, Denmark; 2 Institute of Regional Health Research, University of Southern Denmark, Campusvej 55, 5230 Odense, Denmark; 3 Department of Medicine, Zealand University Hospital, Roskilde, Sygehusvej 10, 4000 Roskilde, Denmark; 4 Department of Emergency Medicine, Hospital of Southern Jutland, Kresten Philipsens Vej 15, 6200 Aabenraa, Denmark; 5 Department of Cardiology, Odense University Hospital, J. B. Winsløws Vej 4, 5000 Odense, Denmark

**Keywords:** cardiovascular disease, chronic kidney disease, acute myocardial, incidence rate

## Abstract

Chronic kidney disease (CKD) is a known risk factor for cardiovascular disease, including acute myocardial infarction. However, whether this risk is only associated with severe kidney disease or is also related to mildly impaired kidney function is still under debate. The incidence rate and risk factors of incident acute myocardial infarction (AMI) in patients with CKD are sparse. Potential differences in risk factor profiles between CKD patients with incident AMI and CKD patients with a prior AMI have not been sufficiently investigated. Furthermore, important factors such as albuminuria and socio-economic factors are often not included. The primary aim of this study was to establish the incidence rate of AMI after CKD debut. Secondly, to evaluate the importance of different CKD stages and the risk of having an AMI. Finally, to identify individuals at risk for AMI after CKD debut adjusted for prevalent AMI. Based on data from the kidney disease cohort of Southern Denmark (KidDiCo), including 66,486 CKD patients, we established incidence rates and characteristics of incident AMI among patients within a 5-year follow-up period after CKD debut. A Cox regression was performed to compute the cause-specific hazard ratios for the different risk factors. The incidence rate for CKD stage G3–5 patients suffering acute myocardial infarction is 2.5 cases/1000 people/year. In patients without a previous myocardial infarction, the risk of suffering a myocardial infarction after CKD debut was only significant in CKD stage G4 (HR = 1.402; (95% CI: 1.08–1.81); *p*-value = 0.010) and stage G5 (HR = 1.491; (95% CI: 1.01–2.19); *p*-value = 0.042). This was not the case in patients who had suffered an acute myocardial infarction prior to their CKD debut. In this group, a previous myocardial infarction was the most critical risk factor for an additional myocardial infarction after CKD debut (HR = 2.615; (95% CI: 2.241–3.05); *p*-value < 0.001). Irrespective of a previous myocardial infarction, age, male sex, hypertension, and a low educational level were significant risk factors associated with an acute myocardial infarction after CKD debut. The incidence rate of AMI in patients with CKD stage G3–5 was 2.5 cases/1000 people/year. Risk factors associated with incident AMI in CKD stage G3–5 patients were CKD stage, age, and hypertension. Female sex and higher educational levels were associated with a lower risk for AMI. Prior AMI was the most significant risk factor in patients with and without previous AMI before fulfilling CKD stage G3–5 criteria. Only age, sex, and a medium-long educational level were significant risk factors in this group.

## 1. Introduction

Chronic kidney disease (CKD) is an increasing global challenge, affecting around 13% of the world’s population [1,2]. Acute myocardial infarction (AMI) is a leading cause of death and disability worldwide [3]. CKD is an independent risk factor for the development of cardiovascular disease (CVD) [4]. Although comprehensive data on AMI prevalence in CKD patients is available (Annual Data Report|USRDS), data on AMI incidence is only available for the general population [5] (HjerteTal (shinyapps.io)). The AMI incidence rate has only been described in CKD patients after orthopedic surgery [6]. To our knowledge, no data on AMI incidence after CKD debut is available.

Kidney Disease: Improving Global Outcomes (KDIGO) has staged CKD into stages G1–5 according to glomerular filtration rate (GFR) [7]. CKD stages G1 and G2 require the presence of kidney damage, e.g., proteinuria. Stages G3–G5 are entirely based on GFR: G3a (GFR = 59–45 mL/min/1.73 m^2^), G3b (GFR = 44–30 mL/min/1.73 m^2^), G4 (GFR = 29–15 mL/min/1.73 m^2^), and G5 (GFR < 15 mL/min/1.73 m^2^). Although CKD is a risk factor for CVD, the relationship between moderately impaired kidney function and CVD has been less clear and needs further investigation [8,9,10,11,12,13]. Previous studies have often measured creatinine; therefore, the results are not directly translatable to CKD stages [9,12,13]. GFR is a more accurate estimate of kidney function; had this been measured, the results from these studies would be more useful.

In addition to CKD being a risk factor for CVD, CVD is independently associated with kidney function decline [14]. CKD and CVD have mutual risk factors, including hypertension (HT) and diabetes mellitus (DM) [15,16,17,18]. Previous studies evaluating the relationship between CKD and CVD have been limited by insufficient adjustment for prevalent CVD AMI [19,20,21]. Yet, a differentiation in terms of risk factors between CKD patients with a prior AMI, and CKD patients with an incident AMI, may yield important information. Low socio-economic status is associated with cardiovascular disease (CV) risk [22]. Lower income and educational levels were associated with a higher prevalence of CKD [23]. However, socio-economic factors are rarely included in publications on CVD risk factors in CKD patients [16].

The primary aim of this study is to establish the incidence rate of AMI after CKD debut. Secondly, to evaluate the importance of different CKD stages and the risk of having an AMI. Finally, to identify individuals at risk for AMI after CKD debut adjusted for prevalent AMI.

## 2. Materials and Methods

The present study included patients from the kidney disease cohort (KidDiCo) database, which consists of data from individuals with CKD living in Southern Denmark between 2006 and 2013. In order to include patients with CKD G3–5 debut only, we excluded CKD G3–5 patients from 2006. Therefore, only patients fulfilling inclusion criteria between 2007 and 2013 with CKD between stages G3 and G5 were included.

The definition of CKD stage G3–5 used in the study was in accordance with the Kidney Disease: Improving Global Outcomes (KDIGO) guidelines, which require two eGFR measurements below 60 mL/min/1.72 m^2^ at least three months apart but not more than one year apart, with no eGFR measurements above 60 mL/min/1.72 m^2^ in the period between [7]. Our cohort has previously used these definitions and has a high coverage rate and representativeness regarding the background population [24,25].

Information about AMI was obtained from the Danish National Patient Register (DNPR) due to the ICD-10 codes I21 (ST-elevation AMI, or STEMI), I21.0–I21.4 (non-ST elevation AMI, or NSTEMI), and DI219 (acute myocardial infarction). In Denmark, patients with AMI are treated exclusively in a hospital setting according to international guidelines [26]. The coding for first-time myocardial infarction in the DNPR has proven highly valid, with a positive predictive value of ≥90% [27]. DNPR data was available from 1977 to 2018.

### 2.1. Variables

KDIGO has staged kidney diseases according to glomerular filtration rate (GFR) and albuminuria. Stage G3 represents a GFR of 59–30 mL/min/1.73 m^2^. KDIGO recommends a subdivision of CKD stage G3 into CKD stage G3a (GFR 59–45 mL/min/1.73 m^2^) and CKD stage G3b (GFR 44–30 mL/min/1.73 m^2^). Stage G4 GFR of 29–15 mL/min/1.73 m^2^ and stage G5 GFR less than 15 mL/min/1.73 m^2^ describe patients where renal replacement therapy might be needed [7]. We included all transplanted patients and patients on hemodialysis in CKD stage G5, regardless of eGFR. The eGFR defined the CKD stage when CKD G3–5 criteria were fulfilled. In the case of transplanted and dialysis patients, the initial ICD-10 code for renal transplantation or dialysis (Z99.2 and Z94.0) was registered, defining the time the patient fulfilled CKD criteria, in this case, CKD stage G5. eGFR was calculated using the Chronic Kidney Disease Epidemiology Collaboration (CKD-EPI) formula recommended by KDIGO, including plasma creatinine, age, and sex [7]. The vast majority of the population was Caucasian, so race was not included.

Demographic data was obtained from the Danish Civil Registration System (DCRS). The DCRS is often used for research and administrative purposes and is subject to ongoing validation and registration by law [28,29].

Albuminuria was divided, according to KDIGO, into three stages. A1 represented albuminuria per creatinine ratio less than 30 mg/g, A2 represented albuminuria between 30 and 300 mg/g, and A3 represented albuminuria above 300 mg/g [7]. The first albuminuria assessment during a 12-month period after fulfilling CKD 3–5 criteria was used to determine the albuminuria stage.

Age, HT, DM, CVD, and socioeconomic factors were registered on the same date as CKD stage 3–5 criteria were fulfilled.

Diagnoses for DM and HT were found in the DNPR and enriched with the following Anatomical Therapeutic Chemicals (ATC) codes +/− 3 months from the time point of CKD incidence: DM: A10 “drugs-used-in-diabetes”; HT: C03 “diuretics”, C07 “beta-blocking agents”, C08 “calcium-channel blockers”; and C09 “agents on the renin-angiotensin system”. CVD was defined as ICD-10 codes for congestive heart failure, peripheral vascular disease, or cerebrovascular disease. Acute myocardial infarction was excluded from CVD and analyzed separately.

Educational levels were divided into short, medium, and long at the time of CKD debut. The short educational levels included primary school, high school, and adult education. Medium education includes a bachelor’s degree or further education at the bachelor’s level. Long education levels include higher education, research, and a PhD. The missing information was stated as such in the table. Data were provided by the Education and Knowledge Register [24].

Employment status is divided into “active” and “not active”. The status “not active” includes pensioners and individuals on welfare. All data are based on the year prior to CKD incidence. This data was provided by the employment classification module [24].

### 2.2. Statistical Analysis

Descriptive statistics were presented as absolute and relative frequencies, as all variables are categorical. In addition, a χ^2^-test was performed to test for differences in relative frequencies between incident AMI patients with CKD stage G3–5 with and without an AMI during the 5-year follow-up period.

We used Cox regression, which is valid in studies investigating competing risks such as death [30]. All exposure variables were included in one Cox regression. Hazard ratios (HR) with corresponding 95% confidence intervals (CI) and *p*-values were computed to evaluate the association between the defined exposures and the hazard for an AMI after CKD diagnosis. A person was censored for not experiencing AMI within the observation period.

The proportional hazard assumption was checked graphically using Schoenfeld residuals. Martingale residuals were not computed as all the predictors were categorical, so the linearity assumption does not apply. Age was divided into three categories: 18–59 years, 60–79 years, and 80 years or more. The division was roughly based on the distribution of AMI in the study population.

Tables and statistical analysis were performed using Stata version 16 [31]. The manuscript was written in accordance with the STROBE statement [32]. The study was approved by the Danish Data Protection Agency (18/36727) and the appointed regional attorneys (20/40602). Furthermore, the study has been reported to the Regional Committee on Health Research Ethics for Southern Denmark (20182000-143).

## 3. Results

**Figure 1:**
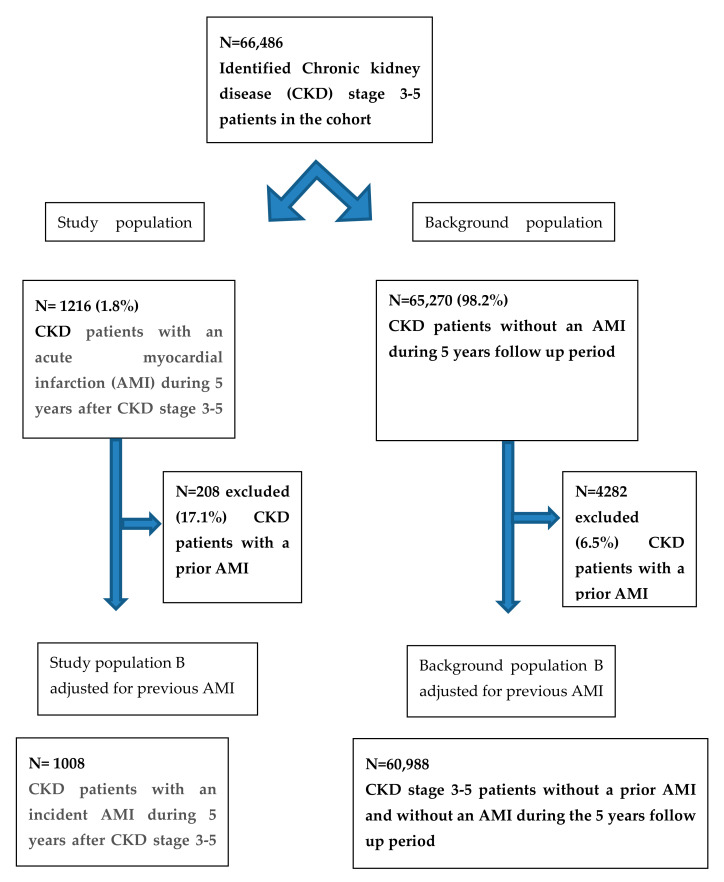
Flow chart showing the selection procedure for the study populations and background populations A and B.

There were 66,486 patients identified with CKD stage G3–5 during the inclusion period of 2007–2013. Of these, 1216 (1.8%) suffered an AMI during the 5-year follow-up period (study population A in Figure 1). After excluding patients with prior AMI, N = 1008 individuals (1.5%) remained who suffered incident AMI after CKD G3–5 debut (study population B in Figure 1). Accordingly, the AMI incidence rate of the 5-year follow-up for CKD G3–5 patients was 2.5 cases/1000 people/year.

**Figure 2:**
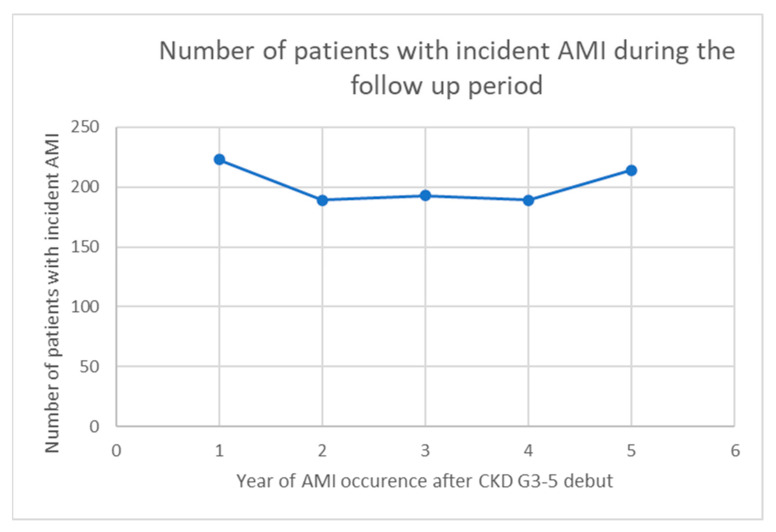
Trends of incident AMI in patients with CKD stage G3–5 during the 5-year follow-up period.

The mean number of incident AMIs was 202 per year (range 189–223). However, the distribution of incident AMIs during the follow-up period did not show any significant trend (see Figure 2).

In the following, we focused on patients with CKD stage G3–5 suffering incident AMI (study population B). The background population consisted of patients who did not suffer an AMI before or during the 5-year follow-up period (background population B in Figure 1).

The percentage of males was lower in the background population (38.0 vs. 50.8%) for all cases (*p* < 0.001) (see Table 1). There was no difference in age between the two groups. At incidence, approximately 90% of patients were in CKD stages G3a and G3b in both groups. The majority of patients in both groups were not assessed for albuminuria. Among DM, HT, and CVD, only HT showed a significant difference (*p* < 0.001) with a high percentage in both the background population (79.7%) and the CKD G3–5 group with incident AMI (84.0%). CKD patients with an incident AMI often had a shorter education (82.8 vs. 76%; *p* < 0.001). There were no significant differences regarding employment status.

The cause-specific Cox regression is shown in Table 2.

Female sex was associated with a reduced hazard ratio for incident AMI after CKD debut (hazard ratio (HR) = 0.567; 95% confidence interval (CI) = 0.50–0.64; *p*-value < 0.001). Age was significantly associated with incident AMI after CKD debut, with a continuously increasing HR across the age groups. Only CKD stages G4 and G5 and not CKD stage 3b at CKD debut showed a significant association with incident AMI (HR = 1.402; CI: 1.08–1.81; *p*-value = 0.010) and (HR = 1.491; CI: 1.01–2.19; *p*-value = 0.042). HT was associated with incident AMI after CKD debut (HR = 1.219; CI: 1.03–1.45; *p*-value = 0.024). Medium educational levels (HR = 0.75; CI: 0.60–0.94; *p*-value = 0.013) were associated with reduced risk of incident AMI after CKD stage G3–5 debut.

Within the five-year follow-up period, 21,183 patients (35%) were censored due to death.

The cause-specific Cox regression, including cases with prior AMI, can be seen in Table 3. A prior AMI was the single most prominent risk factor (HR = 2.615; CI: 2.241–3.05; *p*-value < 0.001). Age, sex (HR = 0.607; CI: 0.54–0.68; *p*-value < 0.001), HT (HR = 1.249; CI: 1.05–1.48; *p*-value = 0.010), and medium educational level (HR = 0.88; CI: 0.641–0.968; *p*-value = 0.024), but CKD stages and CVD excluding AMI were not significant risk factors. Characteristics of patients with CKD G3–5 with and without AMI during the 5-year follow-up period can be seen in Table S1.

## 4. Discussion

Based on a Danish cohort, we calculated the incidence rate and risk factors of AMI after CKD debut. The incidence rate of AMI in patients with CKD stage G3–5 was 2.5 cases/1000 people/year. Risk factors associated with incident AMI in CKD stage G3–5 patients were CKD stage, age, and hypertension. Female sex and higher educational levels were associated with a lower risk for AMI. Prior AMI was the most significant risk factor in patients with and without previous AMI before fulfilling CKD stage G3–5 criteria. Only age, sex, and a medium-long educational level were significant risk factors in this group.

The crude incident rate for AMI in the USA in 2013 was somewhat lower than our study population, with an incidence rate of 1.95/1000/year [5]. However, according to the Danish Heart Foundation (HjerteTal (shinyapps.io)), the rate is 2.12/1000/year for men and 1.63 for women in Denmark, corresponding with the fact that the risk of CVD is generally higher in CKD patients.

The present study reinforces the knowledge that CKD G3–5 patients have different risk profiles for AMI depending on whether or not there has been a prior AMI.

According to Schiffrin et al., patients with CKD stage G3 had a 2–4-fold increased risk of an AMI compared to individuals without CKD, and this risk increased 4–10-fold in stage G4 and 10–50-fold in stage G5 [33]. The median age in the present study population was higher than the study population used by Shiffrin et al. who may have overestimated this risk as they compared CKD patients to a population without a CKD background. However, in this study, we only included patients at CKD stage G3–5 and used a CKD background population. More recent studies report fewer convincing results regarding the AMI risk in lower CKD stages [7,12]. However, this observation was confirmed in our study, where only CKD stages G4 and G5 seemed to be risk factors for future AMI in patients without prior AMI. CKD stage was not a risk factor when addressing patients with CKD stage G3–5 and a history of prior AMI. This finding supports the ARIC study, which reported that a previous AMI was a more prominent risk factor for a subsequent AMI than CKD stages [34].

Although albuminuria is established as a risk factor for CVD [15], the results of our study did not support this. An explanation may be found in the sparse albuminuria testing or the higher significance of other included risk factors.

Diabetes was not a risk factor for AMI in the present study cohort. Due to the high median age, one could argue that diabetic changes had already manifested and did not alter the outcome of our study. Although the prevalence of diabetes is increasing in Denmark, mortality and morbidity in these patients are decreasing [35]. We suggest that improved medical treatment for diabetes and its complications may offer a meaningful explanation. However, hypertension is a risk factor and presents a possible primary target to prevent AMI in patients with CKD, regardless of a prior AMI before the debut of CKD stage G3–5.

Prophylactic treatment with RAAS blockade agents and statins is an evidence-based treatment option, yet it is not always prescribed accordingly [16,36]. Despite introducing the treatment of sodium glucose cotransporter-2 inhibitors for CKD patients after the study was completed, this treatment shows promising results [16]. Agents like these should be started early in treatment due to their cardio- and reno-protective properties. CKD G4–5 patients should be followed by a nephrologist to establish preventive treatments, especially prior to AMI [37]. Unfortunately, referral rates for these patients are low [38].

Our study confirmed that age is a significant risk factor for AMI. Regarding sex, it is noteworthy that although more women fulfilled the CKD criteria, female sex was associated with a lower risk for AMI. [39]. Healthier lifestyles or the protective effect of oestrogen may be an explanation [40].

Although this study indicates that occupational status was not a risk factor, the median age suggests most of the study population had retired at the time of inclusion. Remarkably, medium- to long-term educational levels seem to have a protective influence on AMI after CKD debut for our study population. To our knowledge, this finding has not been described previously. Further studies are needed to investigate how to interpret these results. Possibly, education promotion could have a prophylactic effect on AMI in CKD patients. The amount of missing data under educational level may be due to the high number of older patients who have never completed a formally registered education and, therefore, cannot be categorized.

The strengths of this study include a comprehensive longitudinal study of CKD patients with a 5-year follow-up period and the inclusion of albuminuria and socioeconomic data. In addition, the use of highly valid national Danish databases increases the validity of the results.

Limitations: Our analysis did not include data on body mass index or smoking. Identification of HT was based on the medications commonly used in treating HT. However, as this type of medication can be used in other contexts, for example as a diuretic, a limitation of the study was that the HT definition was a proxy for blood pressure assessment and may have overestimated the number of patients with HT. Furthermore, GFRs are subject to change and may have altered during the 5-year follow-up.

## 5. Conclusions

The incidence rate of AMI after CKD debut is 2.5 cases/1000 people/year. Age, sex, and educational levels are important risk factors. Only CKD stages G4 and G5 and not G3b were associated with significant risk factors for incident AMI in patients with CKD stage G3–5. In individuals with AMI before the debut of CKD stage G3–5 patients, the AMI event prior to the CKD stage G3–5 debut was by far the most significant risk factor. Therefore, clinicians’ primary target to prevent incident AMI should be strategies to avoid the patient reaching stage G4–5. The importance of the educational level needs further investigation. Hypertension seems to be a central risk factor for AMI in patients at CKD stage G3–5 and should also play a central role for clinicians concerning preventative strategies.

## Figures and Tables

**Table 1:** Characteristics of CKD G3–5 patients without AMI prior to CKD debut with and without incident AMI during 5-year follow-up.

		Patients with CKD Stage G3–5 without an Incident AMI during the 5-Year Follow-Up Period	Patients with CKD Stage G3–5 with an Incident AMI during the 5-Year Follow-Up Period	*p*-Value *
N		N = 60,988	N = 1008	
Sex	male	23,200 (38.0%)	512 (50.8%)	<0.001
	female	37,788 (62.0%)	496 (49.2%)	
Age (median)		76 (68–83)	76 (70–82)	0.44
CKD stage, according to GFR	G3a	41,408 (67.9%)	711 (70.5%)	0.17
	G3b	14,155 (23.2%)	204 (20.2%)	
	G4	3841 (6.3%)	65 (6.4%)	
	G5	1584 (2.6%)	28 (2.8%)	
GFR (mean)		47.2 (11.2)	47.6 (10.7)	0.31
CKD stage, according to albuminuria	A1	4465 (7.3%)	115 (11.4%)	<0.001
	A2	987 (1.6%)	20 (2.0%)	
	A3	154 (0.3%)	5 (0.5%)	
	missing	55,382 (90.8%)	868 (86.1%)	
Diabetes	no	50,770 (83.2%)	804 (79.8%)	0.003
	yes	10,218 (16.8%)	204 (20.2%)	
Hypertension	no	12,397 (20.3%)	161 (16.0%)	<0.001
	yes	48,591 (79.7%)	847 (84.0%)	
Cardiovascular disease (excluding acute myocardial infarction)	no	46,813 (76.8%)	757 (75.1%)	0.22
	yes	14,175 (23.2%)	251 (24.9%)	
Educational level	short	46,347 (76.0%)	835 (82.8%)	<0.001
	medium	6273 (10.3%)	82 (8.1%)	
	long	371 (0.6%)	6 (0.6%)	
	missing	7997 (13.1%)	85 (8.4%)	
Occupational status	active	4551 (7.5%)	64 (6.3%)	0.026
	not active	55,803 (91.5%)	941 (93.4%)	
	other/missing	634 (1.0%)	3 (0.3%)	

* values below 0.05 were considered significant.

**Table 2:** Hazard ratio of AMI during 5-year follow-up in CKD G3–5 patients without prior AMI.

	Haz. Ratio	*p*-Value *	[95% Conf. Interval]
Sex (female)	0.567	<0.001	0.50–0.64
Age Group			
18–59 years of age	1		
60–79 years of age	1.659	0.002	1.21–2.28
80 years of age and above	1.883	<0.001	1.35–2.64
CKD stage, according to GFR			
G 3a	1		
G 3b	0.960	0.615	0.82–1.13
G 4	1.402	0.010	1.08–1.81
G 5	1.491	0.042	1.01–2.19
CKD stage, according to albuminuria			
A1	1		
A2	0.788	0.326	0.49–1.27
A3	1.382	0.48	0.56–3.39
missing	0.764	0.011	0.62–0.94
Diabetes			
no	1		
yes	1.084	0.344	0.92–1.28
Hypertension			
no	1		
yes	1.219	0.024	1.03–1.45
Cardiovascular disease (excluding acute myocardial infarction)			
no	1		
yes	1.106	0.177	0.96–1.28
Educational level			
short	1		
medium	0.75	0.013	0.60–0.94
long	0.84	0.671	0.38–1.88
missing	0.792	0.051	0.63–1.00
Occupational status			
active	1		
not active	1.181	0.246	0.89–1.57
other/missing	0.365	0.088	0.12–1.16

* values below 0.05 were considered significant.

**Table 3:** Hazard ratio of AMI during 5-year follow-up in CKD G3–5 patients with and without prior AMI.

	Haz. Ratio	*p*-Value *	[95% Conf. Interval]
Sex (female)	0.607	<0.001	0.54–0.681
Age Group			
18–59 years of age	1		
60–79 years of age	1.505	0.005	1.129–2.005
80 years of age and above	1.658	0.001	1.223–2.247
CKD stage according to GFR			
G 3a	1		
G 3b	0.945	0.429	0.82–1.088
G 4	1.162	0.227	0.911–1.481
G 5	1.345	0.106	0.939–1.926
CKD stage according to albuminuria			
A1	1		
A2	0.982	0.929	0.657–1.468
A3	1.767	0.143	0.824–3.789
missing	0.797	0.019	0.658–0.964
Diabetes			
no	1		
yes	1.093	0.245	0.941–1.27
Hypertension			
no	1		
yes	1.249	0.010	1.055–1.479
Cardiovascular disease (excluding acute myocardial infarction)			
no	1		
yes	1.077	0.264	0.946–1.227
Acute myocardial infarction prior to CKD			
no	1		
yes	2.615	<0.001	2.241–3.05
Educational level			
short	1		
medium	0.788	0.024	0.641–0.968
long	1.004	0.992	0.499–2.019
missing	0.833	0.087	0.676–1.027
Occupational status			
active	1		
not active	1.221	0.134	0.94–1.587
other/missing	0.718	0.403	0.33–1.561

* values below 0.05 were considered significant.

## Data Availability

The data underlying this article were provided by Denmark Statistics with permission. Data will be shared on request with the corresponding author with the permission of Denmark Statistics.

## Supplementary Materials

The following supporting information can be downloaded at: https://www.mdpi.com/article/10.3390/jcdd9110387/s1. Table S1: Characteristics of patients with CKD G3–5 with and without AMI during the 5-year follow-up period.
