# Supplementary material for: Rate and Risk Factors of Acute Myocardial Infarction after Debut of Chronic Kidney Disease—Results from the KidDiCo
Source: J Cardiovasc Dev Dis. 2022 Nov 9;9(11):387. doi: 10.3390/jcdd9110387 (PMC9696870; doi:10.3390/jcdd9110387)
Supplement: Supplementary file 1 [file jcdd-09-00387-s001.zip › jcdd-2017149-supplementary.pdf]

Table S1. Characteristics of patients with CKD G3-5 with and without AMI during the 5-year follow-up period.

		**CKD G3-5 patients without an AMI during the 5-year follow-up period**	**CKD G3-5 patients with an AMI during the 5-year follow-up period**	***p*-Value ***
		N=65,270	N=1,216	
Sex	male	25,666 (39.3%)	639 (52.5%)	<0.001
	female	39,604 (60.7%)	577 (47.5%)	
Age (median)		76 (69-83)	76 (69-81)	0.11
CKD stage according to GFR	3a	43,771 (67.1%)	849 (69.8%)	0.22
	3b	15,503 (23.8%)	262 (21.5%)	
	4	4,286 (6.6%)	73 (6.0%)	
	5	1,710 (2.6%)	32 (2.6%)	
GFR (mean)		47.0407 (11.26436)	47.56311 (10.6039)	0.11
CKD stage according to albuminuria	A1	4,807 (7.4%)	135 (11.1%)	<0.001
	A2	1,061 (1.6%)	29 (2.4%)	
	A3	161 (0.2%)	7 (0.6%)	
	missing	59,241 (90.8%)	1,045 (85.9%)	
Diabetes	no	54,119 (82.9%)	961 (79.0%)	<0.001
	yes	11,151 (17.1%)	255 (21.0%)	
Hypertension	no	12,576 (19.3%)	165 (13.6%)	<0.001
	yes	52,694 (80.7%)	1,051 (86.4%)	
Cardiovascular disease (excluding acute myocardial infarction)	no	49,251 (75.5%)	885 (72.8%)	0.032
	yes	16,019 (24.5%)	331 (27.2%)	
Acute myocardial infarction prior to CKD	no	60,988 (93.4%)	1,008 (82.9%)	<0.001
	yes	4,282 (6.6%)	208 (17.1%)	
Educational level	short	49,657 (76.1%)	1,001 (82.3%)	<0.001
	medium	6,617 (10.1%)	100 (8.2%)	
	long	389 (0.6%)	8 (0.7%)	
	missing	8,607 (13.2%)	107 (8.8%)	
Occupational status	active	4,739 (7.3%)	73 (6.0%)	0.071
	not active	59,867 (91.7%)	1,136 (93.4%)	
	other/missing	664 (1.0%)	7 (0.6%)	

* values below 0.05 were considered significant.
